# Micro- and Nanosized Particles in Nasal Mucosa: A Pilot Study

**DOI:** 10.1155/2015/505986

**Published:** 2015-06-01

**Authors:** Lenka Čábalová, Kristina Čabanová, Hana Bielniková, Jana Kukutschová, Jana Dvořáčková, Kateřina Dědková, Karol Zeleník, Pavel Komínek

**Affiliations:** ^1^Department of Otorhinolaryngology, Head and Neck Surgery, University Hospital Ostrava, 17. Listopadu 1790, 708 52 Ostrava, Czech Republic; ^2^Centre of Endoscopic Skull Base Surgery, University Hospital Ostrava, 17. Listopadu 1790, 708 52 Ostrava, Czech Republic; ^3^Faculty of Medicine, University of Ostrava, Syllabova 19, 703 00 Ostrava, Czech Republic; ^4^Nanotechnology Centre, VŠB-Technical University of Ostrava, 17. Listopadu 15/2172, 708 33 Ostrava, Czech Republic; ^5^Institute of Pathology, University Hospital Ostrava, 17. Listopadu 1790, 708 52 Ostrava, Czech Republic

## Abstract

*Objective*. The aim of this prospective study is to evaluate presence and quantity of micro- and nanosized particles (NPs) and interindividual differences in their distribution and composition in nasal mucosa. *Methods*. Six samples of nasal mucosa obtained by mucotomy from patients with chronic hypertrophic rhinosinusitis were examined. Samples divided into 4 parts according to the distance from the nostrils were analyzed by scanning electron microscopy and Raman microspectroscopy to detect solid particles and characterize their morphology and composition. A novel method of quantification of the particles was designed and used to evaluate interindividual differences in distribution of the particles. The findings were compared with patients' employment history. *Results*. In all the samples, NPs of different elemental composition were found (iron, barium, copper, titanium, etc.), predominantly in the parts most distant from nostrils, in various depths from the surface of the mucosa and interindividual differences in their quantity and composition were found, possibly in relation to professional exposition. *Conclusions*. This study has proven the possibility of quantification of distribution of micro- and nanosized particles in tissue samples and that the NPs may deposit in deeper layers of mucosa and their elemental composition may be related to professional exposition to the sources of NPs.

## 1. Introduction

Nanosized particles (NPs), having submicron size from 10 nm to several hundred nanometers, are ubiquitous in environment [1]. They are composed of carbon (e.g., soot) or metal oxides (iron, chrome, nickel, aluminium, copper, zinc, titanium, etc.) and they are mostly antropogenous (fossil fuel combustion, smoking, welding, road traffic, etc.) [2]. Due to their very small size, they possess different properties from the microparticles of the same material (chemical and physical reactivity and interaction with living cells and organisms) [1].

The main routes of entrance of the NPs into the organism, aside from skin and digestive tract, are airways and lungs. They penetrate tissues, redistribute in organism, accumulate in organs, and induce pathological changes in tissues, while their lysosomal degradation in cells is limited [1, 3]. They induce oxidative stress in cells and, thus, changes in their organelles and genetic information and can also enter the cell nucleus and interact with DNA directly [1, 3–9].

So far, the studies have proven pathological effect of NPs on living cells in vitro or in lungs and digestive tract of rodents in terms of induction of acute and chronic inflammatory changes, while the same material of identical chemical composition but micrometer size was not found to be harmful [4, 7, 10–12]. Other studies focused on presence, distribution, and accumulation of NPs in different levels of airways; this was studied on rodents, as well as on computed models. The computed models show assumed distribution of inhaled NPs in the tissues of airways. It is assumed that their behavior when inhaled is different from that of the larger particles. Ghalali's computed model shows that the most of NPs deposit in the nasal cavity on the anterior-most parts of turbinates as well as microparticles, but the second location of the most abundant deposition differs; the NPs tend to deposit in pharynx, whereas the microparticles deposit in larynx [13, 14].

Although the pathological influence of fine and ultrafine particles including elemental metal particles on lungs is well-known (e.g., occupational pneumoconiosis due to exposure to rare metals) [15], only few clinical studies on nanotoxicology and nanopathology in airways have been conducted so far; for example, Zeleník et al. studied presence and elemental compositions of nanoparticles in human palatine tonsils [16].

One of the main challenges of clinical research focusing on nanotoxicology and nanopathology has been only a limited possibility to quantify and determine the concentration of NPs in the examined tissues, because the principle of their detection in tissues is vastly different from that of larger particles [16]. Mapping of the tissues and statistical analysis of the presence of NPs becomes difficult due to the enormously small scale of the “nanoworld.” The main aim of this prospective study was to design and test a novel method of quantification of micro- and nanosized particles in tissue samples.

Since the nasal cavity and its turbinates are the first barrier filtering the inhaled air, it is probable that the airborne nano- and microparticles would deposit in the mucosa of the nasal cavity, enter its deeper layers, and play a role in chronic inflammatory changes. Since the pathophysiological mechanisms of inflammatory changes in chronic rhinosinusitis are not yet completely clear [17], this study also aims to provide first information on deposition, distribution, and elemental composition of nano- and microparticles in the nasal mucosa and future prospects in further research concerning their possible role in inflammatory changes of the mucosa of upper airways.

## 2. Material and Methods

This prospective study was conducted from September 2013 to March 2014. It was approved by the Institutional Ethics Committee (identifier FNO-ENT-Nanoparticles, 2 RVO-FNOs/2013) and registered at ClincialTrials.gov (identifier NCT02270125). The study was performed in accordance with the Declaration of Helsinki, good clinical practice, and applicable regulatory requirements. Informed consent was obtained from all participants before initiation of any procedure.

Patients aged from 19 to 74 (mean 44) with chronic hypertrophic rhinosinusitis nonresponsive to conservative therapy indicated for endoscopic mucotomy were enrolled in the study. Demographic data and occupational histories were obtained from the patients (Table 1). The tissue samples were processed at the Institute of Pathology and examined by scanning electron microscopy (SEM) and Raman microspectroscopy (RMS) at the Nanotechnology Center.

### 2.1. Biopsy and Sample Preparation

**Figure 1:**
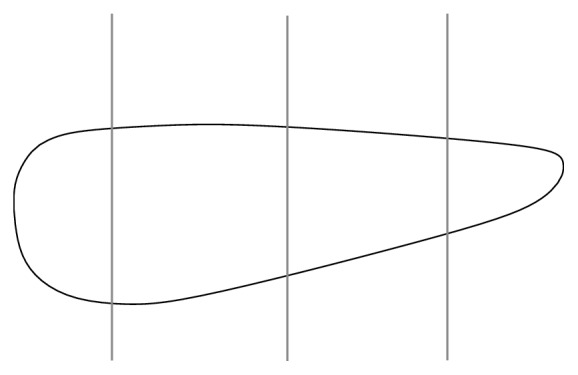
Scheme of a tissue sample obtained by mucotomy; vertical lines indicate division of the sample into four parts according to the distance from the nostrils.

The tissue samples, mucosa of the inferior nasal turbinates, were obtained by endoscopic “cold-steel” mucotomy under general anesthesia. The samples were attached to paraffin tablets by sterile surgical needles; the orientation of the mucosa was marked on the tablet (anterior and posterior sides) and sent to the Institute of Pathology under sterile conditions for further processing. The samples were immersed in 10% formalin and vertically divided into four parts according to the distance from the nostrils: part 1, closest to the nostrils; parts 2 and 3, in the middle; and part 4, closest to choanae (Figure 1). After alcohol-xylene dehydratation and automated paraffin embedding, 2–4 *μ*m thin sections were cut and mounted on glass microscope slides, before staining with hematoxylin/eosin for routine pathological examination. Thin sections were also cut for further chemical analysis (SEM and RMS) and mounted as above. These sections were deparaffinized in xylene and alcohol and were not stained.

### 2.2. Analytical Methods Utilized for Detection and Characterization of Particles

SEM (Quanta FEG 450, FEI) with X-ray microanalysis APOLLO X (EDAX) and SEM Philips XL 30 operating at 30 keV were utilized for morphology characterization and elemental composition of the particles found in single tissue samples. Samples were evaluated in the BSE (back-scattered electrons) mode allowing for visual detection of changes in elemental composition. For example, metallic particles scattering electrons appear as light spots compared to the tissue absorbing electrons and therefore have a dark colour in the BSE mode. Thus, the entire area of each section was inspected and the light spots were analyzed.

**Figure 2:**
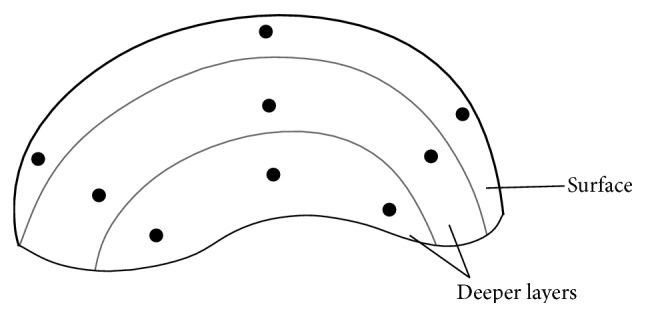
Scheme of cross sections with marked points of Raman microanalysis within superficial and deep layers of the mucosa.

Raman spectra allowing for phase characterization of particles in the human tissue were obtained using a Smart Raman Microscopy System XploRA (HORIBA Jobin Yvon, France), using the 532 nm excitation laser source, 100x objective, and 1200 gr./mm grating in the range from 80 to 2000 cm^−1^. The laser beam with diameter approximately of 0.5 *μ*m allows for point phase analysis of microsized particles. Raman microspectroscopic analysis was performed in each of the tissue sample sections obtained from single sample. To quantify the distribution of particles, a field consisting of 9 points with similar distances from each other and covering the entire sample area was selected in each section (Figure 2, scheme of the cross section). The points of analysis were selected to include the area directly below the nasal mucosa (denoted as superficial layer, approximate thickness of 1 mm) but also areas located in deeper epithelium layers (deep layers, over 2 mm from the surface). Raman spectrum in each selected point was obtained and interpreted.

## 3. Results

In this pilot study, a total of six nasal mucosa samples obtained from six patients with chronic hypertrophic rhinosinusitis were examined. In all the samples, micro- and nanosized particles of different elemental composition were found. Elements detected in individual tissue samples and in their sections according to the distance from the nostrils are listed in Table 2. The most abundant element was iron (also barium, copper, titanium, and zinc).

**Figure 3:**
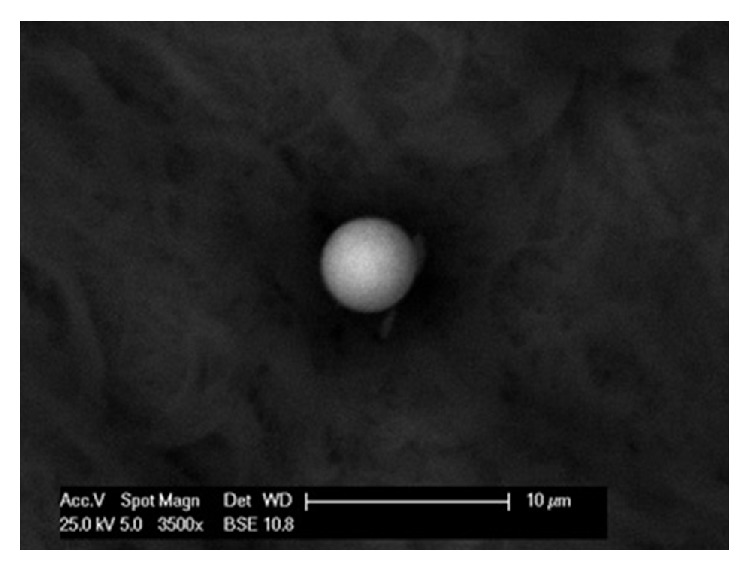
Scanning electron microscope image of a spherical Fe containing particle in the sample A (magnification 3,500x).

**Figure 4:**
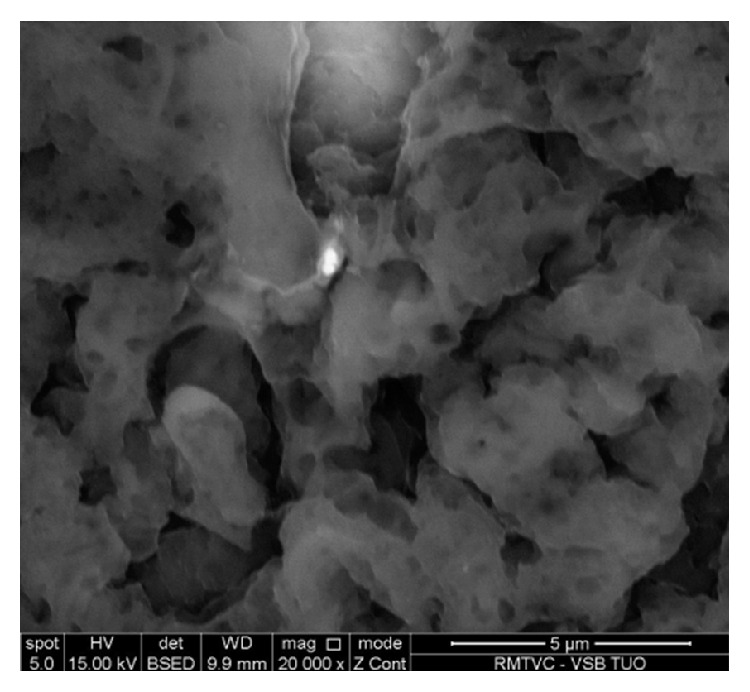
Scanning electron microscope image of an aggregate based on Ba detected in the sample C (magnification 20,000x).

The particles detected by SEM ranged from approximately 10 *μ*m to submicron sizes and most often in form of aggregates/agglomerates and their morphologies varied from spherical to polygonal (Figures 3 and 4). A representative example of a spherical particle composed of metal (Fe) was found in the tissue of a welder (sample A) (Figure 3).

Interindividual differences in elemental composition of particles were found (Table 2). The greatest variety of the detected metals was observed in the tissues of the patients occupationally exposed to welding emissions (samples A and C). Iron in the tissue samples of the welders was present in all sections analyzed.

The samples were analyzed in terms of distribution and quantity of the particles by RMS. Our analysis of the geometry of particle distribution is based on the system of 9-point coordinates as described above. The specific compounds detected by the analysis of 9 points in each tissue section using RMS are listed in Table 3. The highest incidence of the particles in all samples was found in areas further from the nostrils (parts 2–4), while lower numbers of particles were detected in the proximal area, part 1.

Different areas according to their depth from the surface of the mucosa were also assessed by RMS (Table 3). Interindividual differences were also found: in sample A (retired welder), there was a higher number of particles in deeper layers of the tissue than in the superficial layer, whereas in samples C, D, and F (C, welder/locksmith; D, security guard; F, industrial worker), the particles were found predominantly in the superficial layer. In samples B and E (B, programmer and E, student), there was approximately the same number of particles detected in the superficial and deeper layers of the mucosa.

## 4. Discussion

It has been proven that inhaled NPs of various elemental composition cause acute and chronic inflammatory changes in lower levels of respiratory tract and may also induce malignant transformation of cells, but the role in pathologic changes in the mucosa of the upper airways has not been sufficiently studied yet [1, 2, 4, 10, 12, 18, 19]. So far only a few clinical studies have been conducted; for example, Zeleník et al. studied presence and elemental composition in tissues of palatine tonsils [16]. The behavior of NPs in human tissues and immunologic reaction of the organism is still unclear; further understanding of the role of NPs in inflammation induction may contribute to better understanding of the pathophysiological mechanisms of inflammatory changes in the nasal mucosa in chronic hypertrophic rhinosinusitis.

This pilot study was focused on detection of micro- and nanosized particles in the nasal mucosa, their elemental composition, distribution throughout the tissue samples, and comparison of differences in NPs' quantity in different patients. We did not assess histological changes in the mucosa, which is an objective of a subsequent ongoing study conducted in our department. The possibility of quantification of NPs has been only limited so far as the principle of their detection in tissues is vastly different than in larger particles due to the enormously small scale of the “nanoworld” [16]. In our study we present a novel method of quantification of the nano- and microparticles in the nasal mucosa.

The greatest variety of the detected metals was observed in the tissues of the patients occupationally exposed to welding emissions (samples A and C), mostly iron, which was found in all the sections of the samples. This fact is not surprising as the welding fumes contain mostly iron and manganese [20, 21]. Anyway, welders may be exposed to similar airborne pollutants as the general population (road traffic, industrial pollution, and smoking). Also, we have to consider if using stainless steel instruments during surgery may cause artificial contamination of the tissue samples, as all the examined samples contained iron particles. However, artificial contamination would cause presence of the particles only on the surface or in the superficial-most layers of mucosa and cannot explain presence of the particles in the deep layers of the samples.

The morphology of the detected particles was studied by SEM. The particles varied from spherical to polygonal. Spherical iron particles were found in sample A (welder). These are often produced by high-temperature processes and were described as the most abundant particulate emission in welding fumes [21] (Figure 3).

The highest incidence of the particles in all samples was found in areas distant from the nostrils, while lower numbers of particles were detected in the anterior-most parts of the samples. There is no clear explanation for this finding, but the distribution of the airflow in the nasal cavity, the state of the mucosa, and other factors are likely determinants of the deposition of the inhaled particles. This is in contrast with the results of Ghalati et al. (2012), who assumed that most NPs would deposit on the anterior-most parts of the turbinates based on computed modeling [13]. This difference in results could possibly be explained by deposition and redistribution of the particles in the mucosa. Since our cohort is too small to make any conclusions about the NPs distribution, further research in this area is needed.

There is no clear explanation of the interindividual differences in distribution of NPs in areas of the tissue according to their depth from the surface (Table 3). Further research in this area with larger cohorts of patients and also thorough histopathological examination of the tissues is needed.

Although this pilot study has shown some interesting results, we are aware that the examined cohort is too small to make definitive conclusions concerning the NPs composition, distribution, and interindividual differences and to give any possible explanations of our findings. The study was not meant to provide statistical or quantitative evidence as to the prevalence of particles in nasal mucosa, their types, sources, and the health hazard (if any) they may pose. Our main objective was to design and test a novel work flow platform, which could be used to conduct a more comprehensive analysis.

In the future, the aim of our subsequent ongoing study is to analyze more tissue samples including thorough histopathological examination and to compare the findings in the patients with chronic rhinosinusitis with healthy mucosa samples obtained from cadavers.

## 5. Conclusions

This pilot study has proven the possibility of quantification of distribution of micro- and nanosized particles in tissue samples, which had previously been one of the main challenges of the clinical research in nanopathology. It has also shown that these particles may deposit in deeper layers of nasal mucosa and that their elemental composition may be related to professional exposition to their sources (welding fumes). Since the cohort examined in our study is too small, further research in this area is needed to confirm our findings.

## Figures and Tables

**Table 1:** Summary of sex, age, and occupation history of patients.

Sample	Sex	Age	Occupation
A	M	74	Retired, former welder
B	M	34	Programmer
C	M	34	Locksmith/welder
D	M	61	Security guard
E	F	19	Student
F	F	40	Industrial worker

**Table 2:** Elements revealed in a single sections of the nasal tissue samples by SEM-EDX elemental analysis of the visually detected spots (part 1, anterior part, closest to the the nostrils; parts 2 and 3 in the middle; and part 4, posterior part, closest to choanae).

Element	Samples																							
	A				B				C				D				E				F			
	1	2	3	4	1	2	3	4	1	2	3	4	1	2	3	4	1	2	3	4	1	2	3	4
Ba	x	x				x	x		x	x	x	x		x						x				
Cd												x												
Cu				x			x	x	x	x	x	x												
Cr				x						x	x													
Fe	x	x	x	x	x	x		x	x	x	x	x		x	x	x	x	x	x	x	x	x	x	x
Mn			x								x													
Mo	x																							
Ni				x						x	x	x												
Pb									x															
Ti								x	x	x	x	x		x	x									
Zn		x		x				x		x		x												

**Table 3:** Compounds detected in various sections of the tissue samples by the Raman microspectroscopy. The particles were found in both superficial (S) and deeper (D) layers of the tissue samples, with interindividual differences.

Compound	Samples																							
	A				B	C							D				E				F			
	1	2	3	4	1	2	3	4	1	2	3	4	1	2	3	4	1	2	3	4	1	2	3	4
Anatase	D	D				S				S				S		S								
Rutile								D																
Calcite		D			D	S																		
Magnetite								S			S													
Barite																								
Siderite				D												S	S				S			
Graphite		D										S^*^		S		S	D				S			S
Amorphous carbon	D	D	S											S	D									S

^*∗*^Detected twice.
